# Psychosocial characteristics of alcoholic and non-alcoholic liver disease recipient candidates in liver transplantation: a prospective observational study

**DOI:** 10.1186/s12876-021-02032-9

**Published:** 2021-11-29

**Authors:** Masato Shizuku, Hiroyuki Kimura, Hideya Kamei, Shinichi Kishi, Tatsuya Tokura, Nobuhiko Kurata, Kanta Jobara, Atsushi Yoshizawa, Chisato Tsuboi, Naoko Yamaguchi, Midori Kato, Keita Kawai, Makoto Yamashiki, Emi Kanai, Kanako Ishizuka, Norio Ozaki, Yasuhiro Ogura

**Affiliations:** 1 Department of Transplantation Surgery, Nagoya University Hospital, 65 Tsurumai-cho, Showa-ku, Nagoya, 466-8560 Japan; 2 Department of Transplantation and Endocrine Surgery (Surgery II), Nagoya University Graduate School of Medicine, 65 Tsurumai-cho, Showa-ku, Nagoya, 466-8550 Japan; 3 Department of Psychiatry, Nagoya University Graduate School of Medicine, 65 Tsurumai-cho, Showa-ku, Nagoya, 466-8550 Japan; 4 Transplant Coordination Service, Nagoya University Hospital, 65 Tsurumai-cho, Showa-ku, Nagoya, 466-8560 Japan

**Keywords:** Liver transplantation, Alcoholic liver disease, Alcohol dependence, Psychosocial functioning, Personality, Social support

## Abstract

**Background:**

There are long-standing controversies about the transplant indications for alcoholic liver disease (ALD), because of the recognition that ALD is fundamentally self-inflicted. However, it is unclear whether psychosocial characteristics of ALD are different from that of non-alcoholic liver disease (NALD) in the selection of liver transplantation (LT) recipients. We aimed to clarify the psychosocial characteristics of ALD recipients (ALD-R)/ALD recipient candidates (ALD-RC) and NALD recipients (NALD-R)/ NALD recipient candidates (NALD-RC).

**Methods:**

From 2011 to 2019, 75 patients were enrolled in this prospective observational study (ALD-RC, n = 19; NALD-RC, n = 56), LT were carried out as follow; ALD-R, n = 6; NALD-R, n = 52. We evaluated psychosocial characteristics in the preoperative period and 3, 12 months after LT (ALD-R, n = 3/3; NALD-R, n = 28/25). The following scales were used to evaluate psychosocial characteristics: Visual Analogue Scale, Alcohol Use Disorders Identification Test, Hospital Anxiety and Depression Scale, Beck Depression Inventory, Brief Evaluation of Medication Influences and Beliefs, Social Support Questionnaire (SSQ), Temperament and Character Inventory, Parental Bonding Instrument (PBI), the Short Form Health Survey (SF-36).

**Results:**

When evaluating on the basis of abstinence rule, a comparison of ALD-RC and NALD-RC in the preoperative period identified similar patterns of psychosocial characteristics, except that the NALD-RC scored higher on the PBI item “overprotection from mother” (*P* < 0.05). The only significant difference between ALD-R and NALD-R after liver transplantation was in SSQ scores at 3 months.

**Conclusion:**

The psychosocial characteristics of ALD-RC and NALD-RC may be similar when evaluated on the basis of Japan’s abstinence rule. This result also imply that the psychosocial characteristics of ALD-RC may differ from the previously reported psychosocial characteristics of alcohol dependent patients. These findings have the potential to provide helpful information for the evaluation of ALD-RC.

## Background

Liver transplantation for alcoholic liver disease involves many issues, such as the validity of the 6-month abstinence rule [1–3], indications regarding liver transplantation for acute alcoholic hepatitis [4–6] and alcohol relapse [7, 8]. In particular, the importance of psychosocial evaluation before and after liver transplantation to prevent alcohol relapse has been emphasized [9, 10]. It is generally assumed that patients with alcoholic liver disease have previously experienced alcoholic dependence. Therefore, it is very important to manage alcohol relapse in patients who have undergone liver transplantation for alcoholic liver disease. Previous research has shown that postoperative psychological intervention reduces the risk of alcohol relapse after liver transplantation [11–14].

There may be some distinctive problems such as preconceptions that patients with alcohol dependence or alcohol-related diseases are weak-willed. Indeed, we tend to consider those who have received a liver transplant for alcoholic liver disease as alcohol-dependent patients who also have other addictions, depression, and alcohol-related problems. However, the alcohol relapse rate among liver transplant recipients who had alcoholic liver disease is approximately 30–40% [2, 15, 16], whereas this rate is approximately 60–70% among alcohol dependent patients [11, 12]. Despite this evidence, we may be not able to completely distinguish alcoholic liver disease from alcohol dependence when evaluating candidates for liver transplantation for alcoholic liver disease.

There have been many studies on psychosocial evaluations for liver transplant recipients; however, it is unclear whether psychosocial characteristics differ between alcoholic liver disease recipient candidates (ALD-RC) and non-alcoholic liver disease recipient candidates (NALD-RC). To our knowledge, no previous reports have examined such differences in psychosocial characteristics. This study aimed to clarify the psychosocial characteristics of ALD-RC and to inform the appropriate selection of liver transplant recipient candidates.

We performed a prospective observational study that focused on the following three points. First, we compared the psychosocial characteristics of ALD-RC and NALD-RC before liver transplantation on the basis of current abstinence rule in Japan. Second, we evaluated psychosocial characteristics consecutively in alcoholic liver disease recipient (ALD-R) and non-alcoholic liver disease recipient (NALD-R) patients who actually received a liver transplant at 3 and 12 months after liver transplantation. Third, we compared scores on the Medical Outcomes Study 36-Item Short Form Health Survey (SF-36) between ALD-R and published Japanese norms at 3 and 12 months after liver transplantation.

## Methods

### Study population and data collection

In this prospective observational study, we enrolled 75 liver transplant recipient candidates aged 16 years or older (ALD-RC, n = 19; NALD-RC, n = 56) who were referred to our hospital from January 2011 to December 2019. The recipient candidates in this study were patients who met the physical criteria for liver transplantation based on the ethical guidelines of the Japan Society for Transplantation [17, 18].

### The selection process for liver transplant candidates

To conduct appropriate assessment of indications for liver transplantation, our center created a transplantation medical team in 2004. This team comprises transplant surgeons, gastroenterologists, hepatologists, psychiatrists, transplant coordinators, and psychologists. When patients are referred to our hospital, transplant surgeons, gastroenterologists, and hepatologists evaluate whether they meet the indication for liver transplantation such as primary liver disease, blood tests, model for end-stage liver disease score, within Milano criteria and Child–Pugh classification C. In Japan, the transplantation criteria about alcohol use disorder indicates that absolute contraindication only for alcohol use disorders without 6 months of sobriety (changed to 18 months after March 2014), therefore, ALD-RC are required to have followed a 6-month abstinence rule at the initial assessment. Following physical evaluation, transplant coordinators check their family situation and support system. Psychiatrists and psychologists check psychosocial characteristics, namely performance on the Visual Analogue Scale (VAS), Alcohol Use Disorders Identification Test (AUDIT), Hospital Anxiety and Depression Scale (HADS), Beck Depression Inventory (BDI), Brief Evaluation of Medication Influences and Beliefs (BEMIB), Social Support Questionnaire (SSQ; Number of Persons, NP; Satisfaction Rating, SR), Temperament and Character Inventory (TCI), Parental Bonding Instrument (PBI), and the Medical Outcomes Study 36-Item Short Form Health Survey (SF-36; Physical Component Summary, PCS; Mental Component Summary, MCS).

### Postoperative follow-up of liver transplant recipients

The team continues to hold interdisciplinary conferences every week to share patient information [10]. After liver transplantation, all patients received ongoing support from psychiatrists and psychologists and, in cases of comorbid psychiatric disorders, received psychiatric treatment such as pharmacotherapy or psychotherapy.

### The aim in this study

In this study, we conducted the following three investigations. First, on the basis of current abstinence rule in Japan, we compared psychosocial characteristics, namely scores on the VAS, AUDIT, HADS, BDI, BEMIB, SSQ (NP/SR), TCI, PBI, and SF-36 (PCS/MCS), between ALD-RC and NALD-RC before liver transplantation. Second, we evaluated psychosocial characteristics, namely scores on the VAS, AUDIT, HADS, BDI, BEMIB, SSQ (NP/SR), SF36 (PCS/MCS), at the preoperative period and at 3 and 12 months after liver transplantation for patients who received liver transplantation. Third, we compared SF-36 scores between ALD-R and published Japanese norms at 3 and 12 months after liver transplantation. Japanese norms were obtained from data reported by Fukuhara et al., “Manual of SF-36 Japanese version 1.2” [19–21].

Although we cannot rule out selection bias, we hoped to avoid this as much as possible by identifying all patients diagnosed within a specific period of time.

### Measurements

The **VAS** assesses subjective characteristics or attitudes that cannot be directly measured. Scores range from 0 to 100 points [22, 23]. We measured pain in this study. The **AUDIT** is a measurement tool to screen patients for possible alcoholism. The AUDIT comprises a 10-item questionnaire and is a simple and useful method for early detection of harmful alcohol use [24]. The **HADS** scale assesses levels of anxiety and depression. The Japanese version of the HADS questionnaire was used to assess patients’ depression severity [25, 26]. Higher scores indicate more severe symptoms. The **BDI** is a self-rating measurement tool developed by Beck et al.; it has high diagnostic utility for depression [27, 28]. The **BEMIB** was developed to identify non-adherence in patients with psychiatric disorders and consists of an 8-item scale. In this study, we used the Japanese version of the BEMIB [29]. The **SSQ** measures social support and satisfaction with social support from the perspective of the interviewee. The SSQ consists of two subscales: the Number of Persons (NP) subscale, which reflects the sum of the perceived number of others who provide social support, and the Satisfaction Rating (SR) subscale, which reflects the sum of the individual’s degree of satisfaction with perceived social support. In this study, the six-item Japanese version of the SSQ was used [30, 31]. The **TCI** assesses personality characteristics. According to Cloninger [32, 33], personality can be divided into temperament (Novelty Seeking, Harm Avoidance, Reward Dependence, Persistence) and character domains (Self-directedness, Cooperativeness, Self-transcendence). Participants respond to each TCI item by answering “true” or “false” and a total score for each temperament and character dimension is calculated. In this study, we used the Japanese version of the TCI [34]. The **PBI** assesses perceived rearing [35]. The PBI is a self-report questionnaire that evaluates a person’s perception of how they were raised by asking the respondent to recall their parents’ child-rearing attitudes before the age of 16 years. The PBI consists of four categories: paternal care, paternal overprotection, maternal care, and maternal overprotection, and scores are obtained for each category. In this study, we used the Japanese version of the scale [36]. The **SF-36** is a 36-item patient-reported survey of patient health. Administration of the SF-36 generates a health profile on eight domains: general health, physical function, role physical, bodily pain, vitality, social functioning, mental health, and role emotional. The SF-36 also provides two higher-order summary scores: the physical component summary (PCS) and the mental component summary (MCS). Scores for each summary range from 0 to 100. Higher scores indicate better health-related quality of life [37]. Published Japanese norms were used as a comparison for SF-36 scores. We compared recipients’ health as measured by the SF-36 with national standards using these norms [19–21].

### Statistical analysis

Statistical analyses were performed using SPSS, version 25 (IBM, Armonk, NY, USA). Continuous variables were compared by *t* test and Mann–Whitney U test.

Homogeneity of variance was evaluated using Levene’s test. For between-group comparisons, Student’s t-test was used if the data showed homogeneity of variance. In the absence of homogeneity of variance, Welch’s method was used. The chi-square test was used to examine differences between categorical variables. A *P*-value of < 0.05 was regarded as statistically significant. We did not determine an a priori sample size; owing to the small pool of study subjects, we conducted the analysis using the maximum sample size available. We compensated by checking the effect size after the analysis. Because of the exploratory nature of this study, we did not adjust for multiple comparison issues. A statistical review of the study was performed by a biomedical statistician. Because of the exploratory nature of this study, we did not adjust for multiple comparison issues. A statistical review of the study was performed by a biomedical statistician.

## Results

**Fig. 1:**
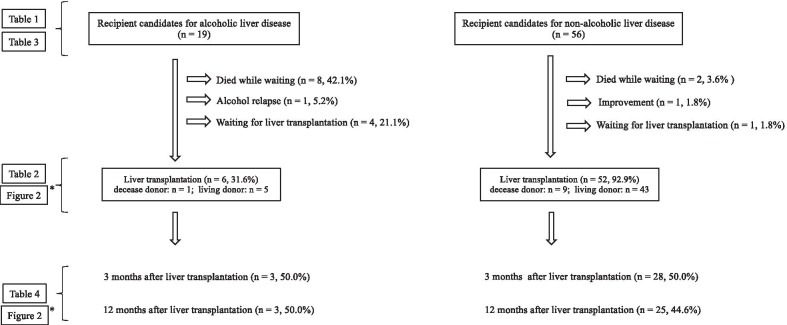
Flow chart of participants enrolled in this study. Data were responses to questionnaires that were mailed to all subjects and returned to us. Statistical analysis was performed after excluding missing data. *Compared with published Japanese norms

The ALD-RC who met the physical criteria for liver transplantation were 76 patients at initial assessment, however, 19 patients were evaluated because 57 patients were excluded as ALD-RC by pre-liver transplantation evaluation (Shortage of absent drinking or fail to quit drinking; 32, Improvement of liver function after initial evaluation; 2, Drop-out of follow-up before liver transplantation; 2, Withdraw offer for liver transplantation; 2, Transfer to other hospital; 2, Development of other diseases; 1, Unknown; 16). Additionally, in the 76 ALD-RC who met the physical criteria, 27 patients were applied the 18-month abstinence rule (before February 2014) and 49 patients were the 6-month abstinence (after March 2014). Finally, as shown in Fig. 1, there were 19 and 56 evaluable patients in the ALD-RC and NALD-RC groups, respectively. Of the 19 ALD-RC, six (31.6%) actually underwent liver transplantation (living donor liver transplantation: 5; deceased donor liver transplantation: 1), eight (42.1%) died waiting for a transplant, one (5.2%) experienced alcohol relapse, and four (21.1%) continued to wait for liver transplantation. One patient was excluded from liver transplantation because of alcohol relapse. This patient was a 38-year-old man who had been treated for alcoholic cirrhosis for 5 years. On physical and psychosocial evaluation, he appeared to meet the evaluation criteria for liver transplantation but was subsequently found to have had a small number of alcohol relapses, which prompted a reevaluation. This man did not receive support from a psychiatrist or psychologist before liver transplantation because he lived far away from the studies hospital. Of the 56 NALD-RC, 52 (92.9%) actually underwent liver transplantation (living donor liver transplantation: 43, deceased donor liver transplantation: 9), two (3.6%) died waiting for a transplant, one (1.8%) experienced improvement, and one (1.8%) was still waiting for liver transplantation. The numbers of patients evaluated at 3 months after liver transplantation in the ALD-R and NALD-R groups were 3 and 28 patients, respectively. At 12 months after liver transplantation, three ALD-R and 25 NALD-R were evaluated.

The demographic characteristics of the ALD-RC and NALD-RC groups are shown in Table 1. There was no significant difference in any of the variables. History of psychiatric disorders also showed no significant differences between ALD-RC and NALD-RC. The primary diseases experienced by the ALD-RC are shown in Table 2. The perioperative characteristics of the ALD-R and NALD-R who underwent liver transplantation are shown in Table 3. There was no significant difference between the two groups in any of the variables.

**Table 1:** Demographic characteristics of the ALD-RC and NALD-RC groups

Variables	ALD-RC group (*n* = 19)	NALD-RC group (*n* = 56)	*P*-value
Age (mean years, range)	47.7 (37–60)	46.8 (16–67)	0.82
Sex (Male/Female)	11/8	26/30	0.29
AST (IU/L) (mean, SD)	52.1 (28.1)	247.8 (1085.0)	0.44
ALT (IU/L) (mean, SD)	27.8 (15.0)	184.6 (763.5)	0.39
Hepatic encephalopathy (point, mean, SD)	1.2 (0.4)	1.3 (0.5)	0.25
Ascites (point, mean, SD))	1.8 (0.8)	2.0 (0.9)	0.48
Total Bilirubin (mg/dL) (mean, SD)	7.7 (7.3)	8.6 (10.9)	0.76
Albumin (g/dL)	2.9 (0.5)	2.8 (0.7)	0.58
Prothrombin time (%) (mean, SD)	46.5 (17.2)	57.1 (20.9)	0.10
PT-INR (mean, SD)	1.61 (0.4)	1.51 (0.6)	0.69
Creatinine (mg/dL) (mean, SD)	0.84 (0.4)	1.07 (1.3)	0.49
MELD score (point) (mean, SD)	18.1 (6.2)	18.1 (10.2)	0.93
Child–Pugh score (point) (mean, SD)	10.3 (1.7)	9.9 (2.3)	0.55
*Primary liver disease*			
Number of HCC (%)	5 (26.3)	5 (8.9)	0.11
Psychiatric disorders (Present/Absent)	8/11	25/31	0.85

ALD-RC: Alcoholic liver disease recipient candidate; ALT: Alanine transaminase; AST: Aspartate transaminase; HCC: Hepatocellular carcinoma; NALD-RC: Non-alcoholic liver disease recipient candidate; MELD: Model for end-stage liver disease score; PT-INR: International normalized ratio of prothrombin time; SD: Standard deviation

**Table 2:** The primary disease of the ALD-RC and NALD-RC

	ALD-RC (n = 19)	NALD-RC (n = 56)
*Primary liver disease*		
Alcoholic liver cirrhosis	14	0
Alcoholic liver cirrhosis with HCC	5	0
*Primary sclerosing cholangitis*	0	9
Primary biliary cholangitis	0	8
Biliary atresia	0	5
Non-alcoholic steatohepatitis	0	5
Autoimmune hepatitis	0	4
Fulminant hepatitis	0	4
Hepatic C virus related cirrhosis	0	3
Polycystic liver disease	0	3
Hepatic B virus related cirrhosis with HCC	0	3
Hepatic B virus related cirrhosis	0	2
Hepatic C virus related cirrhosis with HCC	0	2
Wilson disease	0	1
Hepatic fibrosis	0	1
Idiopathic portal hypertension	0	1
Congenital biliary dilatation	0	1
Hypoplastic right hepatic lobe	0	1
Unknown	0	3

ALD-RC, Alcoholic Liver Disease Recipient Candidate; HCC, Hepatic Cell Carcinoma; NALD-RC, Non-Alcoholic Liver Disease Recipient Candidate

**Table 3:** A comparison of perioperative characteristics for ALD-R and NALD-R groups

Variables	ALD-R group (*n* = 6)	NALD-R group (*n* = 52)	*P*-value
Age (mean, range)	52.3 (46–60)	46.5 (16–67)	0.31
Sex (Male/Female)	3/3	24/28	0.87
MELD score (point) (mean ± SD)	20.0 ± 8.6	17.9 ± 10.6	0.65
Operation time (min) (mean ± SD)	698 ± 53	757 ± 157	0.39
Blood loss (ml)	10,615 (4468)	9295 (8353)	0.77
Donor type (Decease/Living)	1/5	9/43	0.98
Graft type (left/right/whole)	1/4 /1	4/40/8	0.78
Blood type (identical/compatible/incompatible)	5/0/1	31/ 13/8	0.37
Hospital stay (day) (mean, SD)	79.0 (37.7)	83.1 (79.9)	0.90
Follow up period (day) (mean, range)	1160 (478–2150)	1626 (107–3874)	0.25
*Postoperative outcomes*			
Alive (%)	5 (83.3)	47 (90.4)	0.61
Dead (%)	1 (16.7)	5 (9.6)	
*Postoperative complications*			
Clavien-Dindor classification Grade ≥ IIIb (%)	3 (50.0)	16 (30.8)	0.39

ALD-R: Alcoholic liver disease recipient; MELD: Model for end-stage liver disease score; NALD-R: Non-alcoholic liver disease recipient; SD: Standard deviation

The comparison of psychosocial characteristics in the ALD-RC and NALD-RC groups before liver transplantation is shown in Table 4. The only significant between-group difference was for “overprotection from mother”; the NALD-RC group scored higher on this characteristic.

**Table 4:** Comparison of psychosocial characteristics between ALD-RC group and NALD-RC group before liver transplantation

Variables	ALD-RC	NALD-RC	t	df	*P*	r
	*n* = 19	*n* = 56				
	Mean (SD)	Mean (SD)				
VAS*	14.28 (18.32)	15.52 (20.52)	–	–	0.87	
AUDIT	3.67 (6.06)	1.65 (3.62)	1.64	62	0.11	
HADS	9.83 (7.51)	9.60 (6.59)	0.12	58	0.90	
BDI	11.44 (9.49)	9.07 (7.11)	1.07	59	0.29	
BEMIB	28.33 (5.49)	28.44 (4.80)	− 0.07	45	0.95	
*SSQ*						
NP	4.11 (1.84)	4.82 (2.97)	− 0.94	60	0.35	
SR	5.24 (0.81)	5.09 (1.22)	0.47	58	0.64	
*TCI*						
Novelty Seeking	8.63 (2.73)	8.10 (2.57)	0.67	53	0.50	
Harm Avoidance	11.38 (4.95)	11.26 (4.04)	0.09	53	0.93	
Reward Dependence	9.31 (1.99)	10.11 (2.13)	− 1.27	51	0.21	
Persistence	2.24 (1.92)	2.78 (1.53)	− 1.03	25	0.31	
Self-Directedness	14.19 (4.46)	16.09 (5.14)	− 1.27	47	0.21	
Cooperativeness	17.47 (3.63)	18.42 (3.21)	− 0.98	53	0.33	
Self-Transcendence	3.67 (2.95)	3.62 (3.05)	0.06	55	0.95	
*PBI*						
Care/Father	15.31 (8.20)	11.97 (9.62)	1.22	53	0.23	
Over-protection/Father	29.00 (5.65)	29.66 (6.73)	− 0.34	52	0.73	
Care/Mother	12.94 (8.34)	8.08 (9.31)	1.90	56	0.06	
Over-protection/Mother	23.41 (7.39)	28.71 (7.69)	− 2.39	53	**0.02**	0.31
*SF-36*						
PCS	26.84 (21.35)	28.68 (17.85)	− 0.30	47	0.77	
MCS	49.80 (12.68)	44.65 (11.34)	1.36	47	0.18	

Bold value indicate* p* < 0.05

ALD-RC: Alcoholic liver disease recipient candidate; AUDIT: Alcohol Use Disorders Identification Test; BDI: Beck Depression Inventory; BEMIB, Brief Evaluation of Medication Influences and Beliefs; HADS: Hospital Anxiety and Depression Scale; NALD-RC: Non-alcoholic liver disease recipient candidate; PBI: Parental Bonding Instrument; SF-36: Medical Outcomes Study 36-Item Short Form Health Survey (PCS: Physical Component Summary; MCS: Mental Component Summary); SSQ: Social Support Questionnaire (NP: Number of Persons; SR: Satisfaction Rating); TCI: Temperament and Character Inventory; VAS: Visual Analogue Scale

*Mann–Whitney t test was used to evaluate the differences between two groups

Table 5 shows the results of the between-group comparisons in scores on the VAS, AUDIT, HADS, BDI, BEMIB, SSQ (NP/SR), and SF-36 (PCS/MCS) at 3 and 12 months after liver transplantation for ALD-R and NALD-R. The only significant difference between the two groups was for SSQ (NP) score at 3 months after liver transplantation; the ALD-R group scored higher than the NALD-R group on the SSQ (NP). However, there were small number of patients in this result, especially in ALD-R group (3 months, ALD-R: 3, NALD-R; 28; 12 months, ALD-R: 3, NALD-R: 25). Therefore, although this result may be reference, caution is warranted in interpreting these results.

**Table 5:** A comparison of ALC-group and NALD-R group at 3 and 12 months after liver transplantation

Variables	3 months after liver transplantation			12 months after liver transplantation		
	ALD-R	NALD-R	*p*	ALD-R	NALD-R	*p*
	n = 3	n = 28		n = 3	n = 25	
	Mean ± SD	Mean ± SD		Mean ± SD	Mean ± SD	
AUDIT	0.00 ± 0.00	0.46 ± 1.25	0.54	0.00 ± 0.00	0.33 ± 1.27	0.66
HADS(T)	3.67 ± 3.22	9.46 ± 7.31	0.19	2.00 ± 2.65	7.64 ± 7.33	0.20
BDI	8.67 ± 7.51	9.52 ± 8.03	0.86	4.67 ± 4.04	8.68 ± 7.66	0.39
BEMIB	33.33 ± 4.16	31.79 ± 4.34	0.56	34.00 ± 3.46	34.09 ± 3.04	0.96
*SSQ*						
NP	9.50 ± 6.30	4.67 ± 3.52	**0.04**	5.06 ± 1.01	4.35 ± 2.08	0.57
SR	5.67 ± 0.58	5.06 ± 1.06	0.35	5.08 ± 0.12	5.17 ± 0.93	0.89
*SF-36*						
PCS	17.47 ± 28.25	26.03 ± 16.63	0.47	37.42 ± 10.93	43.80 ± 12.55	0.41
MCS	57.33 ± 14.03	54.22 ± 9.91	0.62	58.65 ± 6.77	53.77 ± 9.31	0.39

Bold value indicate* p* < 0.05

ALD-RC, Alcoholic Liver Disease Recipient Candidate; AUDIT, Alcohol Use Disorders Identification Test; BDI, Beck Depression Inventory; BEMIB, Brief Evaluation of Medication Influences and Beliefs; HADS, Hospital Anxiety and Depression Scale; NALD-RC, Non-Alcoholic Liver Disease Recipient Candidate; SF-36, The Medical Outcomes Study 36-item short form Health Survey (PCS, Physical Component Summary; MCS, Mental Component Summary); SSQ, Social Support Questionnaire (NP, Number of Persons; SR, Satisfaction Rating)

**Fig. 2:**
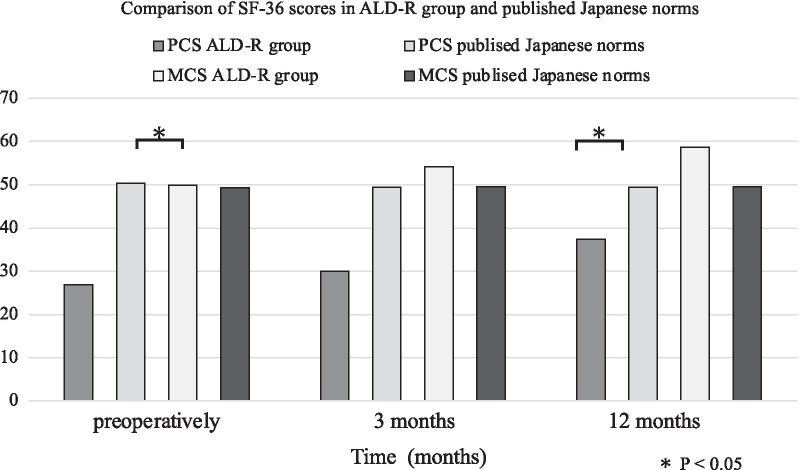
Comparison of SF-36 scores in ALD-R group and published Japanese norms. Although PCS scores in ALD-R were significantly lower than published Japanese norms preoperatively and at 12 months after transplantation, there was no significant difference in MCS scores at any time. ALD-R: Alcoholic liver disease recipient; MCS: Mental component summary; PCS: Physical component summary; SF-36: Short Form Health Survey

Figure 2 shows the comparison of SF-36 (PCS/MCS) scores between ALD-R and published Japanese norms. Although the PCS score in the ALD-R group was significantly lower than published Japanese norms preoperatively and 12 months after liver transplantation, there was no significant difference in MCS score at any time. These results indicate that the ALD-R had significantly poorer physical health compared with national standards, not only preoperatively but also 12 months after liver transplantation. In contrast, there was no significant between-group difference in mental health at any time.

## Discussion

ALD is one of the indications for liver transplantation, and the psychosocial evaluation of patients with alcoholic liver disease is extremely important. However, it is unclear whether psychosocial characteristics differ between ALD-RC and NALD-RC. The present research is the first prospective observational study to examine the psychosocial characteristics of recipient candidates in detail. In this study, there were few psychosocial differences between ALD-RC and NALD-RC. These findings indicate that the psychosocial characteristics of ALD-RC and NAD-RC may be similar when evaluated on the basis of Japan’s abstinence rule. Moreover, the findings also imply that the psychosocial characteristics of ALD-RC may differ from the previously reported psychosocial characteristics of alcohol dependent patients, such as unfavorable maternal bonding [38, 39]. Therefore, there is a need to recognize these differences and evaluate ALD-RC after confirming whether an abstinence rule is applied or not.

Some medical workers have the preconception that alcohol-dependent patients are weak-willed and non-compliant and that their illness is self-inflicted. However, previous studies have reported a considerable difference in the rate of alcoholic relapse between liver transplant recipients with alcohol liver disease [2, 15, 16] and alcohol-dependent patients [11, 12]. The present findings demonstrate almost identical psychosocial characteristics between the ALD-RC and NALD-RC groups when applying either the 6-month or the 18-month abstinence rule. There are five explanations for this similarity. First, the pre-referral of recipient candidates for liver transplantation by previous doctors may have resulted in a selection effect by excluding patients with very poor psychiatric symptom control. Second, the patients had a sufficient and enthusiastic family support system. Third, the patients’ alcohol abstinence may have been prompted by others’ expressions of disgust during their frequent treatment or hospitalization. Fourth, although the patients may have been heavy drinkers, they may not have been alcohol dependent. Fifth, alcohol dependence factors in ALD-RC may have been corrected through the abstinence rule. It is also possible that the psychosocial characteristics of ALD-R and NALD-RC may be similar, but we cannot conclude this because of the small sample size.

In this study, 57 of the 76 ALD-RC met the physical criteria, however, more than half of these patients (32 of 57) were excluded because of alcohol-related problems. Moreover, eight patients in the ALD-RC group died waiting for a transplant. In Japan, the 18-month abstinence rule for liver transplantation for alcoholic cirrhosis was applied through February 2014, and a 6-month abstinence rule has been used since March 2014. Without these abstinence rules, the patients in the ALD-RC group might have had more opportunities for liver transplantation. However, because the ALD-RC and NALD-RC were similar in terms of psychosocial characteristics, the length of abstinence rule in the present study may have made the psychosocial characteristics of the ALD-RC different from those typically seen with alcohol dependence, such as poor medication adherence or alcohol relapse [2, 15, 16, 40–42]. In this study, it is unclear whether an abstinence rule is appropriate, but evaluators should consider the situation in terms of alcohol-related diseases and the number of donors in own country.

Notably, the social context of each county (e.g., alcohol-drinking behavior and the nature of the medical system) should be considered when interpreting the implications of this study. Previous studies in Western countries have reported that alcoholic liver disease is associated with parental neglect and abuse [40–42] and that acloholic liver disease is comorbid with certain psychiatric disorders [43]. Our study showed different results, finding no significant differences in psychosocial characteristics or in history of psychiatric disorders between ALD-RC and NALD-RC at initial assessment. No previous studies have specially investigated this issue, but prior work has demonstrated that over-protection from supporters/ partners is more common in patients with physical diseases such as diabetes mellitus or myocardial infarction [44, 45]. The paradoxical results in our study may have been caused by events such as those described above or by elements of Japan’s unique social context. Additionally, as shown in Fig. 1, the percentage of patients who died while waiting for liver transplantation differed for ALD-RC (42.1%) and NALD-RC (3.6%). In Japan, approximately 85% of liver transplantations have been reported to be from living donors because of a lack of deceased donors [46]. Therefore, patients without potential donors in their families do not have the opportunity for liver transplantation. Because liver donation is voluntary, it is possible that fewer family members offered to serve as donors for patients in the ALD-RC group than for those in the NALD-RC group. Additionally, the Japanese transplantation criteria regarding alcohol use disorder may further reduce the chance of receiving liver transplantation from a deceased donor for those with alcoholic liver disease. Thus, the specific local contexts should be considered when interpretating this article.

In this study, we also compared the ALD-R group’s performance with published Japanese norms for the PCS and MCS. Our results showed that the PCS score in the ALD-R group was significantly lower than published Japanese norms, not only preoperatively but also 12 months after transplantation. However, there was no significant difference in MCS score at any time. Previous studies have reported that PCS scores are lowest preoperatively and then gradually recover beginning in the first month after transplantation, and that MCS scores recover rapidly beginning 1 month after transplantation [47, 48]. Our results are similar in that we also found that PCS and MCS scores gradually recovered from 3 months after transplantation.

Our study has several limitations. One study limitation is that we did not directly compare the psychosocial characteristics of ALD-RC and alcohol-dependent patients; therefore, further research is needed. Another limitation is that this research was a single-institution prospective observational study using data on a small group of patients. The number of patients who underwent liver transplantation was especially small (ALD-R: 3, 3 and NALD-R: 28, 25 in 3 and 12 months after liver transplantation, respectively), therefore, we could not draw definitive conclude whether the psychosocial characteristics of ALD-R and NALD-R are really similar, and we did not examine whether our results are unique to our institution or similar in other institutions or countries. Future multicenter collaborative research is needed to investigate these issues. In addition, we should also consider that the alcohol abstinence rule has changed from March 2014, that is, the 6-month or 18-month abstinence rule. This change might affect the number of patients who received liver transplantation or the results in this study.

## Conclusion

The psychosocial characteristics of ALD-RC and NALD-RC may be similar when evaluated on the basis of the abstinence rule. This result also implies that the psychosocial characteristics of ALD-RC may differ from the previously reported psychosocial characteristics of alcohol-dependent patients. These important findings indicate that we must appropriately evaluate both ALD-RC and NALD-RC, and our results have the potential to provide helpful information for the evaluation of ALD-RC.

## Data Availability

The datasets used and analyzed during the current study are available only upon request, as the data contain potentially identifying or sensitive psychosocial information(alcohol abuse, childhood experience, personality trait, history of psychiatric disorders and so on). All the data supporting our findings are contained within the manuscript.

## Abbreviations

ALD: Alcoholic liver disease
LT: Liver transplantation
ALD-R: ALD recipients
ALD-RC: ALD recipient candidates
NALD-R: Non-ALD recipients
NALD-RC: Non-ALD recipient candidates
SSQ: Social Support Questionnaire
NP: Number of Persons
SR: Satisfaction Rating
PBI: Parental Bonding Instrument
SF-36: The Medical Outcomes Study 36-Item Short Form Health Survey
PCS: Physical Component Summary
MCS: Mental Component Summary
VAS: Visual Analogue Scale
AUDIT: Alcohol Use Disorders Identification Test
HADS: Hospital Anxiety and Depression Scale
BDI: Beck Depression Inventory
BEMIB: Brief Evaluation of Medication Influences and Beliefs
TCI: Temperament and Character Inventory
